# A Manual of the Diseases of Infants and Children at the Breast

**Published:** 1845-10

**Authors:** 


### ART. VI.

*Manuel Pratique des Maladies des Nouveaux-Nés, et des Enfants à la Mamelle, etc.* Par E. BOUCHUT, D.M. etc.—*Paris,* 1845.

*A Manual of the Diseases of Infants and Children at the Breast.* By E. BOUCHUT, M.D.—*Paris,* 1845. 12mo, pp. 616.

MOST of the treatises on the diseases of children which we receive from our neighbours on the other side of the channel are the productions of the industry of young men during the short time of their stay in the large hospitals of Paris. Hence we often meet in them with interesting observations on morbid anatomy, sometimes with the lucid detail of symptoms, but rarely with remarks of much value on the treatment of disease. The work of M. Bouchut differs from most of these, in that it derives its chief value not from his own researches, but from its embodying the results of the experience of his master, M. Trousseau. The Hôpital Necker, to which M. Trousseau is physician, presents a field of observation different in some respects from that of the Foundling Hospital at Paris, where most French writers on the diseases of early infancy have acquired their knowledge; and more like that afforded by ordinary practice. Many of the children in the Hôpital Necker are suckled by their own mothers, and have not been exposed to the same injurious influences as beset the inmates of the Foundling Hospital. Those of our readers who see the French journals must have frequently noticed the valuable clinical lectures of M. Trousseau, and cannot fail to have been struck with the acuteness of observation which they display. We have heard it reported that he is preparing a work on the diseases of children; but while waiting for it, we gladly receive such an earnest of what may be expected from him as is afforded by M. Bouchut's Manual. A little more labour on the author's part would, we think have made it a better book, and though he speaks in his preface of "numerous observations," we confess that we do not always meet with such traces of them as we could have desired. It is, nevertheless a good and useful work; one which we can safely recommend as a valuable addition to the medical library of any of our readers who feel interest in the investigation of children's diseases.

The first part of the work, comprising nearly a hundred pages, is largely, rather too largely, borrowed from M. Donné's excellent little book on the 'Physical Education of Children.' In the second part, which consists of General Observations on the Diseases of Early Infancy, we find much that is valuable, and meet, for the first time, with traces of M. Trousseau. We should have liked, however, to have been furnished with some means of distinguishing between statements which are vouched for by the teacher, and those for which the pupil only is responsible. Thus, at p. 96 we find the assertion that the yellow colour of new-born infants, *icterus neonatorum,* is not due to jaundice, but depends on the slow absorption of the blood infiltrated into the tissues at birth. In support of this opinion he states that the conjunctiva in these cases does not present a yellow tinge. In this assertion, however, he is certainly mistaken; and not only this circumstance, but likewise the influence of cold in inducing and aggravating it; the association of icterus with induration of the cellular tissue; the influence which purgatives exert in removing the yellow colour of the skin; and, above all, the fact that not one half of all new-born children are affected by it,—may serve to invalidate his theory.

The expression of the features, the attitude and gestures, the cry, and the various conditions of the abdomen and mouth, are successively examined; and the author then comes to the respiration, and to the results of examination in the chest. Some remarks are made on the character of the respiration in early infancy, which we have already met with in a lecture by M. Trousseau, and which are to a great extent correct. After noticing the variable character in the respiration in early infancy, and the consequent importance of auscultating children under various circumstances, M. Bouchut alludes to the generally received opinion as to the puerility of the respiration of all young children.

"This statement," says he, "may be correct in the case of children of two years of age; indeed, we have often satisfied ourselves of its truth; but it is erroneous if asserted of the new-born infant, or of children at the breast. Respiration is in them neither sonorous nor noisy; it is attended with a sound of slight intensity, quite destitute of all mellowness, but a kind of coarse breathing which cannot possibly be referred to a complete dilatation of the air-vesicles. We have studied this character of the respiration with extreme care. We have repeated our examination every day, and have never heard anything at all analogous to puerile respiration. This fact is easily explained by the difficulty with which the air enters the lung; either on account of the density of the organ or of the minuteness of the pulmonary vesicles. The density of the lung diminishes with the advance of age, while at the same time the diameter of the vesicles increases; two circumstances, each of which is favorable to the production of puerile breathing.

"There exists a close connexion between puerility of respiration and sonoriety of the thorax. These two phenomena coexist, and if the one ceases, the other disappears. This is readily explicable, since increased tenuity of the pulmonary substance is the cause to which both are due.

"The chest of children at the breast is but slightly sonorous; a fact of which any one may easily satisfy himself. The resonance, however, varies much even in a state of health. It is very slight in fat and healthy children, and more considerable in such as, though healthy, are naturally thin. It changes much and presents singular alternations in the same child and within a very short space of time, independent of any disturbance of the health. Thus, if we percuss the chest for some minutes, an alternate increase and diminution of its resonance become very perceptible; an increase during inspiration, a diminution in expiration. This phenomenon is very marked during the extensive respiratory movements which may be observed in children when crying. Its explanation is easy; the sound is clear during inspiration, that is to say when there is much air in the chest; it is dull in expiration when almost the whole of the air has been expelled." (pp. 121-2.)

To much of this we subscribe, though the facts appear to us rather overstated. The muscular system of the young infant is comparatively little developed, and to the want of the efficient cooperation of the respiratory muscles, it is probably in great measure owing that the lungs are less fully distended than in after-life:—not to any peculiar density in the structure of the lung, nor to the smallness of the pulmonary vesicles impeding the entrance of air. We believe this circumstance, though not noticed by M. Trousseau or his pupil, to be the chief cause of the peculiarity of the respiration in the young child. If, after listening to an infant's chest while breathing gently, as it does at the breast, we again apply our ear during one of those long inspirations which follow a fit of crying, we shall hear the same kind of puerile breathing as in an older subject, although less intense, owing to the smaller size of the lung. In weakly children the respiratory muscles do not lend their aid to carry on the process of breathing; the lung, by its natural resiliency, contracts, and air not being inspired with force adequate to dilate it, it passes into that state of carnification, the true nature of which has been recently pointed out by MM. Bailly and Legendre.

After some remarks on the respiration in a pathological state, there follow notices of the circulation in infancy. M. Roger's recent and most elaborate researches on this subject may dispense us from entering on an examination of M. Bouchut's observations. He next passes, by a natural transition from investigations into the natural frequency of the circulation, to the subject of fever and febrile reaction in children at the breast, the characters of which he traces briefly, but excellently well. He points out how insufficient acceleration of the pulse is to mark the existence of fever, since the pulse ranges in early infancy between very wide extremes, quite independently of any morbid condition. Acceleration of the pulse, however, and heat of skin, when combined with signs of general *unwellness*, indicate its approach. In many points it differs from fever in the adults, since the tongue seldom becomes coated with thick fur, but usually continues moist. Shivering does not occur, but the stage of rigor is supplied by a condition in which the face grows pale, the lips become livid, and the tips of the fingers acquire a blueish tint; and even these symptoms are not observed in the fever which ushers in inflammatory attacks. The temperature next rises two or three degrees, and perspiration breaks out, but never so abundantly in the infant as in the adult: it is a moisture of the skin rather than a copious sweat; often indeed so slight as to be scarcely perceptible. The febrile disturbance that attends acute affections is by no means uniform in intensity: it falls, then rises again, and many paroxysms occur in the course of a single day. In chronic affections the intermittent character of the fever is still more remarkable.

With these remarks on fever the second part of the work closes, and the author enters on the third and last part, in which he treats of the individual diseases. These are classified according to organs,—an arrangement probably as good as any more complicated mode of classification. This part opens with the subjects of dentition considered in its physiological and pathological relations. Some interesting observations are made on the connexion of diarrhea with dentition, from which we make the following extract:

"Of 110 children cutting their teeth, 26 did not suffer from indisposition of any kind, 38 were restless, had stomach-ache, and purging which continued for a very short time, while 46 had abundant diarrhea. In 19 of these latter diarrhea came on whenever the gums became swollen, and ceased when their swelling abated, recurring with the appearance of each tooth, while nothing of the kind was observed in the intervals. In the remaining 28, in whom the process of dentition was very laborious, the diarrhea was persistent and assumed by degrees the characters of inflammatory diarrhea, terminating in enterocolitis." (p. 196.)

Even when this diarrhea is mild it is desirable to check it, since it may otherwise become habitual, and give rise to intestinal disease. In entering on the subject of diarrhea M. Bouchut makes some very good observations on the too great tendency to localize the affection in every instance, and to refer it to some form or other of intestinal lesion. He distinguishes 1st. That form of diarrhea which results from simple acceleration of the peristaltic movement of the intestines. 2d. That which depends on increased secretion of intestinal mucus; or catarrhal diarrhea. 3d. That which results from inflammatory affection of some part or other of the intestines; inflammatory diarrhea, or entero-colitis. His account of inflammatory diarrhea is one of the most valuable chapters in the book. Entero-colitis is one of the most formidable affections of early infancy; to which period of life it is almost limited. The alterations of the intestine to which it gives rise commence in the large intestine, and by their extension affect the small intestine; a relation just the opposite to that which exists in typhoid fever. The large intestine is usually contracted, owing to the spasm of the muscular coat, while its mucous membrane is thrown into folds, the summit of which present evident marks of inflammation. It is of a red colour of varying intensity, interspersed with little prominent white bodies with a central depression, which are the hypertrophied mucous crypts. The whole membrane is sometimes of an uniform red colour; at other times this hue exists only at the summit of the intestinal folds, where erosion of the surface first appears. The ulcerations are usually narrow, shallow, sinuous, corresponding to the summit of the folds, and may often be overlooked unless the intestine be examined against the light. While these attack the summit of the folds, small, superficial, circular, ulcerations form around the muciparous crypts, but in chronic cases are often found cicatrized, a slight depression only marking their situation. Unless the disease run a chronic course, there is seldom much diminution in the thickness of the mucous membranes, but it is usually considerably softened, so that on any attempt to detach it, it comes away in small fragments.

In the acute stage the submucous cellular tissue is rarely affected. When the disease, however, runs a chronic course this tissue becomes slightly thickened, white, indurated, and sometimes semitransparent. These changes seem to take place just at the time when the spasm of the muscular tunic is causing contraction of the intestine. Thus is produced a non-extensile envelop around a contracted organ which is forcibly compressed, and prevented from returning to its former size. There are no changes of moment in the muscular tissue of the large intestine, and the alterations in the small intestine seldom exceed some degree of congestion with tumefaction and softening of the mucous membrane for a short distance from the ileocecal valve.

It is a disease insidious in its onset, obstinate in its course, and often fatal in its result. And no children are so liable to it as those who are of feeble constitution, or exhausted by previous illness or destitution. Restlessness, causeless cries, irritability of the stomach without loss of appetite, and slight diarrhea, with yellow homogeneous stools, are the earliest symptoms; none of which would lead to the suspicion of any graver affection than catarrhal diarrhea. The child, however, loses flesh, sometimes with great rapidity; it seems ill, low spirited, and in pain. It often refuses the breast, or vomits the milk mixed with bilious matter; and this regurgitation of the contents of the stomach, with efforts at vomiting, returns several times a day. The mouth becomes by degrees dry, red, ulcerated, or coated with aphthæ; the diarrhea increases, and the child passes ten or fifteen motions daily, which lose their yellow colour and assume various appearances; becoming likewise sometimes highly acid. Notwithstanding the severity of these symptoms, the fever which attends them is seldom severe, and after the first two or three days presents distinct remissions. The exacerbations recur at indefinite periods, and the heat of skin during them is sometimes very considerable as well as the acceleration of the circulation. The rise of temperature, however, is far from being invariably synchronous with the increased rapidity of the pulse. If the disease terminate fatally, the fever by degrees puts on a typhoid character; if it pass into the chronic state, the fever becomes distinctly intermittent; its accessions usually taking place twice in the twenty-four hours, but never at regular intervals. The abdominal symptoms remain much the same, but emaciation becomes extreme before death, previous to which the fever generally resumes the continued type.

In hospital practice, the mortality from this disease is very great; more than half of those who are attacked by it dying. Out of an hospital, however, the prognosis is far more favorable.

In many cases great benefit will result from changing the nurse, if the child be still at the breast, or from restoring to it the breast if it had been weaned at an early age. In addition to these and other dietetic measures, M. Trousseau has used emetics of ipecacuanha with great success. The formula he employs is the following:

Ipecacuanha (0·30 to 0·60 grammes) gr. 4½ to gr. 9.

Simple syrup (40 grammes) = ʒx.

Half to be taken by a child from 1 to 2 years of age, and to be repeated in ten minutes.

This dose may be repeated on the following morning, if it should not have produced much effect, but it would not be prudent to carry its employment further.

The acid smell of the breath, or acid reaction of the stools, indicates the employment of the various absorbents. Among astringents, both tannin and nitrate of silver are used by M. Trousseau; the latter in the following form:

| | | |
|---|---|---|
| Nitrate of silver, | 1 centigramme | = about gr. 1-6th. |
| Distilled water | 30 grammes | = ʒvii, gr. 43. |
| Syrup . | 10 grammes | = ʒij, gr. 34. |

Many of these medicines too may be employed in enemata; as 30 or 50 centigrammes of tannin to 150 or 200 grammes of fluid; or 1 or 2 centigrammes of alum, or 5 centigrammes of nitrate of silver. Opium appears to be sparingly used by M. Trousseau, if we may judge from the caution with which the author alludes to its employment. We find, however, to a great extent, the same fault in this as in other French works of a professedly practical nature, namely, that there is no clear exposition of the different conditions under which different remedies become indicated. Such works leave the young practitioner in the position of a person introduced into a large armoury, wherein he sees weapons of all kinds, many of them strange and unknown, and all nearly equally useless, owing to the absence of any one who could explain how each is to be employed.

Passing over several chapters which appear less important, we come at p. 314 to an elaborate article on pneumonia, which presents some points for criticism. In his remarks on lobular pneumonia, M. Bouchut takes no notice of the interesting observations of MM. Bailly and Legendre on this condition, which they show to be, in the greater number of instances, wholly independent of any inflammatory process, and the result of a return of the lung to its fetal condition, the vesicles emptying themselves of the air, under certain circumstances, of which they give a minute description. A portion of lung which has thus become solid, dark, and apparently condensed, will, like that of a child who has never breathed, assume a perfectly natural appearance if inflated. Though he takes no notice of these observations, however, M. Bouchut repeats some startling assertions with reference to the influence of inflation of the air-tubes upon inflamed lung. These statements, which he originally made in his inaugural dissertation, and which were controverted by MM. Bailly and Legendre, are to the effect that lung in the second or third degree of hepatization may always be distended with air by insufflation, when it will again become crepitant, and float on the surface of water. He now repeats this assertion, though somewhat less positively; he allows that lung in the state of gray hepatization cannot always be thus distended. He reiterates his former statement, however, with reference to lung in the stage of red hepatization, but he gives no details of experiments, without which so strange an assertion will scarcely obtain much credence. We have repeated the experiments several times, and have found that, in the case of a lung being simply congested, inflation will restore the bright colour, and increase the sense of crepitation, but that no such result is produced on lung which is really in a state of inflammatory hepatization. Like MM. Bailly and Legendre, we have found the carnified or fetal condition of the lung in combination with a congested state of the pulmonary vessels, and, in such a case, insufflation will produce exactly those effects which M. Bouchut has described. This state, however, is one very different from true red hepatization, though the author seems not to have sufficiently distinguished between them.

His general description of the symptoms and course of pneumonia is good, though it does not present anything remarkable. We may notice, however, one or two signs on which M. Trousseau partly founds his prognosis in this and in some other diseases, especially as they serve to illustrate the habit of observation which we have noticed as possessed in so remarkable a degree by that physician.

"There are some symptoms," says the author, "whose importance as prognostics seems to have attracted M. Trousseau's attention, who forms a very correct opinion from indications of this sort. The distension of the veins of the hand for instance, like œdema in the adult, coincides with some impediment to the circulation. According to him the sign is of ill omen when present in pneumonia; it shows that a considerable obstacle exists to the aeration of the blood; or in other words that the disorganization of the lung is very extensive.

"The same holds good with reference to tears. This indication of suffering in a child in health, ceases so soon as it becomes ill. The child cries, but the secretion of tears no longer takes place, and they do not begin to flow again till a considerable improvement has taken place in the condition of the patient. This sign deserves then to be taken into consideration: it exists in all the acute diseases of infancy, and in pneumonia among the rest, but is not observed in chronic affections." (p. 338.)

When treating on the subject of hooping-cough, the author notices a fact which, though not unobserved before, had never been insisted on so much as its importance deserves till M. Trousseau directed attention to it. It is that the supervention of any febrile affection in the course of hooping-cough almost always diminishes, sometimes suspends or completely cures the cough, though exceptional cases do occur in which, notwithstanding the supervention of some such affection, the cough continues unalleviated.

M. Bouchut has collected some facts illustrative of points in the history of hooping-cough concerning which we have but little trustworthy information. Of 33 children affected with hooping-cough 24 had previously suffered from catarrh; in 2 it was absent; and in the remaining 7 it could not be ascertained whether or no it had existed. Paroxysms of cough occurred in twenty-four hours after the commencement of the catarrh in 2 cases; in 5 two days after; 1 three days; 4 five days; 1 six days; 1 seven days; 2 eight days; 1 twelve days; 1 fifteen days; 1 seventeen days; 1 twenty-four days; 2 thirty days; 1 sixty days; and in one case the time was unknown. Eight of the 33 died, 12 left the hospital uncured, 10 got well in the hospital, and one after leaving it. In one of those who recovered, the hooping-cough lasted 3 days, in two 11 days, in one 14, one 19, one 20, and in one 23 days; in one it continued for a month, in another for 7 weeks, in one for 3 months, and one did not recover till after the lapse of 10 months. Some other results are elicited from the above data, but the numbers are so small that it is hardly worth while to do more than refer our readers to p. 379, where they will find all details given in full.

The section on cerebral disease opens with some interesting remarks on convulsions, from which we make the following extract:

"We have collected 41 cases of convulsions of children at the breast in 27 of which the cases were idiopathic, in 14 symptomatic. Fifteen of the children, in whom the convulsions were idiopathic, were attacked by them in the midst of perfect health, and recovered without any ill result; 4 died several months afterwards of other diseases, and an examination did not disclose any important changes in the brain. In 12 the convulsions occurred in the course of other diseases which were serious from their commencement, or at the close of pneumonia, or in the course of erysipelas, or of the fever that attends the development of the vaccine vesicle, and 7 of them died. Only 1 of them, however, presented any morbid appearance in the brain, which consisted in the presence of a tubercle surrounded by unchanged cerebral substance, in the centrum ovale of Vieussens on the right side. This summary is very interesting; it shows most positively that convulsions may occur, 1st, in the midst of perfect health; 2d, during the course of acute affections, in which it seems to be analogous to delirium; 3d, that there does not exist any relation between convulsions of certain parts, and particular tissues of the nervous centres; since it appears from our autopsies, that the encephalon of 10 out of 11 children who died at different periods after convulsive seizures presented no morbid appearance whatever.

"The cases of symptomatic convulsions were caused six times by granular meningitis, twice by simple meningitis, four times by encephalitis with and without tubercles, once by real, idiopathic, acute, hydrocephalus, and lastly in one instance by cerebral tubercle without inflammation of the brain." (p. 387-8.)

The frequency of convulsions in children is shown by the fact that of 16 infants who chanced to be at the same time in M. Trousseau's ward, 7 had previously suffered from convulsions, and the affection was hereditary in the family of several. A remarkable case is related by the author, in which 9 out of a family of 10 children had suffered from convulsions at some period or other, of which 6 had died. He notices other causes of their occurrence besides hereditary tendency; but they are chiefly such as all persons are familiar with. The irritation of over-purgation, however, on which M. Trousseau insists as a cause of convulsions, is too often overlooked, owing to the widely-diffused prejudice which regards convulsions as almost invariably associated with a constipated state of the bowels.

From the consideration of idiopathic convulsions the author passes to those forms of convulsions which are the result of cerebral disease. This introduces him to the subject of granular or tubercular meningitis, of which disease he gives an extremely good description. The account of tubercle of the brain is rather meager, and, with the exception of a case of idiopathic acute hydrocephalus (the word being used in its strict sense), detailed at p. 445, there is nothing particularly noteworthy in the remainder of his observations on cerebral disease.

Remarks on vaccination, and a description of an epidemic of measles which prevailed at the Hôpital Necker in 1843, are succeeded by a brief notice of some skin diseases. The last sixty pages of the work contain an account of various affections not treated of under other heads, among which, however, we are surprised to find no mention of the different forms of the tuberculous cachexia to which children are liable, the chapter on tubercular meningitis, and a scanty notice of cerebral tubercle including all that he says on that most important class of diseases.

---

